# Opening the black box of agency in contraceptive decision-making: A cross-country qualitative study of a complex process

**DOI:** 10.1016/j.ssmqr.2025.100615

**Published:** 2025-12

**Authors:** Lauren Suchman, Emily Himes, Catherine Birabwa, Sneha Challa, Sarah Okumu, Zachary Kwena, Louisa Ndunyu, Serah Gitome, Pauline Wekesa, Mandayachepa Nyando, Martha Kamanga, Tamandani Jumbe, Innocencia Mtalimanja, Alfred Maluwa, Lynn Atuyambe, Dinah Amongin, Betty Kaudha, Agnes Kayego, Ivan Idiodi, Chioma Okoli, Aminat Tijani, Ayobambo Jegede, Awawu Grace Nmadu, Shakede Dimowo, Janelli Vallin, Beth Phillips, Elena Sinha, Address Malata, Elizabeth Bukusi, Peter Waiswa, Elizabeth Omoluabi, Kelsey Holt

**Affiliations:** a Institute for Health & Aging, University of California San Francisco, UCSF Institute for Health & Aging, Box 0646 490 Illinois St., Floor 12, San Francisco, CA, 94158, USA; b Department of Family and Community Medicine, University of California San Francisco, 995 Potrero Ave, San Francisco, CA, 94110, USA; c Makerere University School of Public Health, New Mulago Gate Rd, Kampala, Uganda; d Centre for Microbiology Research, Kenya Medical Research Institute, Mbagathi Rd, Nairobi, Kenya; e Department of Public Health, Maseno University, Busia Road Private Bag 407 05, Kisumu, Kenya; f Academy of Medical Sciences, Malawi University of Science and Technology, P.O. Box 5196, Limbe, Malawi; g Midwifery Department, Kamuzu University of Health Sciences, 52X8+782, Mahatma Gandhi Rd, Blantyre, Malawi; h AkenaPlus Health, 20F Road, Citec Estate, Along Ring Road, Mbora, Abuja, Nigeria; i Family Health Care Nursing Department, University of California San Francisco, 490 Illinois Street, San Francisco, CA, 94143, USA; j Departments of Global Health and Obstetrics and Gynecology, University of Washington, 1410 NE Campus Pkwy, Seattle, WA, 98195, USA; k Department of Obstetrics, Gynecology and Reproductive Sciences, University of California San Francisco, 1825 Fourth St., San Francisco, CA, 94158, USA; l Global Public Health, Karolinska Institute, Stockholm, 171 77, Sweden; m Busoga Health Forum, P.O. Box 805, Jinja, Uganda

## 1 Introduction

Researchers in the family planning field have long puzzled over what makes women choose contraception or not when they do not actively desire pregnancy. Historically, the focus of these studies has been on predictors or determinants of contraceptive uptake with the intention of creating or improving programs to maximize contraceptive use (Ahinkorah et al., 2020; Tesfa et al., 2022). However, recent shifts in the field point to the importance of privileging women’s human rights in the contraceptive decision-making process. Researchers following these shifts put women’s agency—the capacity to think and act in alignment with their preferences—and autonomy— the freedom to make independent choices—first regardless of whether or not they ultimately choose to use contraception (Galavotti & Gullo, 2022; Hardee et al., 2014a, 2014b; Senderowicz, 2020). Indeed, a focus on uptake assumes that using contraception is the right choice for all women not actively seeking pregnancy, although some women may deliberately choose not to use contraception for a variety of reasons that align with their own priorities and preferences (Holt et al., 2023). Further, this body of research has largely treated contraceptive decision-making as a staticprocess that takes place at one moment in time, rather than at multiple points throughout a woman’s life. As Morison and Eager note, the literature also focuses narrowly on the individual level (i.e. behaviors and beliefs) while failing to explore and understand women’s experiences “within the wider social context in which contraceptive practices and decision-making occurs” (Morison & Eagar, 2021). Despite evidence that suggests contraceptive decision-making is a complex process that evolves over a woman’s lifetime (Ralph et al., 2020; Taragin and -Zeller, 2019), there has been little examination of the ways in which this process plays out, including how women exercise agency by actively weighing key influences against each other to reach a final decision that suits their needs. Better understanding how women exercise agency or not can tell us what kind of support is most likely to lead women to reach truly agentive contraceptive decisions.

Particularly over the past decade, researchers have been exploring how reproductive agency can best be defined and measured to enhance global programming (Edmeades et al., 2018; Senderowicz, 2020; Upadhyay et al., 2014). However, many agency-related frameworks or measures focus on romantic partner relationships with less acknowledgment of the broader social context that may enhance or constrain agency. For example, parents may be especially salient for young women’s agency, particularly when these women are unmarried. Holt et al. (Holt et al., 2024) define contraceptive agency as “the ability of an individual to make and act on decisions related to whether to do something to avoid or delay pregnancy and what, if anything, to do when they are not actively trying to become pregnant,” and developed the Contraceptive Agency Framework to delineate elements of agency in contraceptive decision-making and the contextual factors influencing them. The Framework is based on empowerment and health behavior theories (Donald et al., 2020; Montano et al., 2015; Watts et al., 2011), and aims to capture the extent to which people are conscious of their rights and societal injustices, and to broaden the acknowledgment and measurement of sources of interference in agentive decision-making. The Framework also questions key principles taken for granted in much of the literature on contraceptive decision-making, such as equating covert contraceptive use with disempowerment and equating contraceptive use with empowerment (MEASURE Evaluation, 2020; Moreau et al., 2020). It is divided into two domains: 1) Agency in Decision-Making Related to Avoiding or Delaying Pregnancy, and 2) Agency in Acting on Decisions Related to Avoiding or Delaying Pregnancy.

The existing research has demonstrated an association between education, socioeconomic status, and contraceptive use; women who are more educated and wealthier are more likely to use contraception than their counterparts (Blackstone et al., 2017; Demissie et al., 2022; Yaya et al., 2018). Women also are influenced by fears related to contraceptive side effects (Chola et al., 2023) and location (i.e. urban versus rural) often determines the contraceptive products and services women have access to, as well as the likelihood that they face social pressure to use contraception or not (Bogale et al., 2011). Indeed, social context plays a critical role in determining women’s contraceptive use. Social expectations related to women’s sexuality and childbearing in marriage often influence decisions about contraceptive uptake and continuation (Binuyo, 2023; Namasivayam et al., 2022). This is especially true for young women and adolescents, who are subject to conflicting messages that pregnancy outside of marriage is forbidden while contraceptive use is also stigmatized (Chola et al., 2023; Engelbert et al., 2021; Harrington et al., 2021). Such conflicting messages leave this population with few socially acceptable options. Furthermore, much evidence suggests that male partner influence plays an outsized role in women’s contraceptive use. While direct disapproval from male partners is a common barrier to use (Olakunde et al., 2019), prevailing patriarchal norms that privilege men as head-of-household can be just as powerful in preventing women from taking up or continuing contraception. For example, many health facilities in Nigeria require a husband’s permission for his wife to access contraception (Sinai et al., 2020), while some evidence from Kenya shows that providers alter their behavior when working with women who request contraception in order to minimize potential conflict between spouses (Britton et al., 2021). However, such strong influence has the potential to make men powerful allies in contraceptive decision-making, and evidence suggests that men can support their partners' contraceptive uptake and use when adequately informed and sensitively engaged (Kriel et al., 2019; Nkwonta & Messias, 2019).

As the field of family planning moves from focusing on contraceptive uptake to rights-based decision-making constructs, it is important to better understand the *process* of contraceptive decision-making so that quantitative measures can accurately capture women’s experiences, and contraceptive services and programs can be tailored to support agency in the decision-making process, regardless of the outcome. In this analysis of women’s contraceptive decision-making across four countries in sub-Saharan Africa, we sought to deeply understand how women make contraceptive decisions, and the factors that contribute to their ability to have agency in the decision-making process. Specifically, we sought to characterize: 1) key elements of the process of contraceptive decision-making among women in sub-Saharan Africa; 2) within each of these parts of the decision-making process, how women’s ability to exercise agency in decision-making is constrained or bolstered. We use Domain 1 of the Contraceptive Agency Framework (Holt et al., 2024), Agency in Decision-Making Related to Avoiding or Delaying Pregnancy, to structure our discussion.

## 2 Methods

Data were collected and analyzed as part of the Innovations for Choice and Autonomy (ICAN) study. Launched in late 2019, ICAN aims to understand how self-injection of the contraceptive subcutaneous depot medroxyprogesterone acetate (DMPA-SC) can be implemented in a way that best meets women’s needs, as defined by women themselves. ICAN research consortium partners are based at: the Malawi University of Science and Technology (MUST) in Malawi; the Makerere University School of Public Health (MakSPH) in Uganda; the Kenya Medical Research Institute (KEMRI) and Maseno University in Kenya; AkenaPlus Health in Nigeria; and the University of California San Francisco (UCSF) in the United States.

### 2.1 Data collection

From January–September 2021, ICAN teams based in Malawi, Uganda, Kenya, and Nigeria conducted a total of 241 semi-structured, in-depth interviews (approximately 60 interviews per country) with sexually active women aged 15–45 (sampled according to age: 15–19, 20–45) who were both users (n = 151) and non-users (n = 90) of contraception. We defined contraceptive users as anyone who had ever used any method, including “traditional” methods, to prevent pregnancy. Non-users had never used any form of contraception regardless of their desire to prevent pregnancy or not. Study sites included Ntchisi and Mulanje districts, Malawi; Oyam and Mayuge districts, Uganda; Nairobi and Kisumu metropolitan areas, Kenya; and Enugu and Plateau states, Nigeria. Research assistants (RAs) affiliated with the local ICAN consortium partner in each country and fluent in the relevant local language(s) recruited study participants through various methods, such as working with local community health workers and members of local ICAN Community Advisory Boards to identify potential study participants. After obtaining informed consent from participants, RAs audio recorded each interview with the participant’s permission. Interviews were conducted in person following local COVID-19 safety guidelines regarding social distancing and masking. Venues were chosen per participant preference and RAs ensured that interview venues adhered to local COVID-19 safety guidelines while also taking care to preserve participant privacy and confidentiality. Interviews lasted an average of 60 min and included questions about what women consider when making and acting on decisions regarding contraceptive use, including who in their lives (including husbands/romantic partners, family members, healthcare providers, and peers, among others) influences these decisions; women’s perceptions of their own power and other women’s power to make and act on contraceptive decisions; and their previous experiences with and perspectives on contraceptive use.

### 2.2 Analysis

Interview audio recordings were translated and transcribed by teams in each country who were fluent in both the relevant local languages and English. Researchers from each ICAN team performed quality checks on a subset of transcripts before transcripts were uploaded into Dedoose, a qualitative analysis software package. A small group of researchers from each ICAN country team then participated in an in-depth qualitative analysis training before working together to establish standardized code application. This involved using a centralized codebook that was developed deductively based on the interview guide and relevant literature, including the Contraceptive Agency Framework (Holt et al., 2024), and iteratively refined. Following best practices for qualitative analysis (Deterding & Waters, 2021; O'Connor & Joffe, 2020), individual teams from Kenya, Uganda, Malawi, and Nigeria worked to achieve internal code standardization with researchers from the U.S.-based team facilitating and ensuring consistent code application across countries. For this stage of the analysis, each team met as a group and coded a full transcript together over a series of 2-h virtual sessions during which they were able to discuss specific applications of each code. Team members then worked in pairs to code a second transcript and compare their coding choices. If questions arose during this process, teams were able to consult internally, as well as with the ICAN lead of qualitative research. After achieving standardized coding, each participating researcher individually coded a set of assigned transcripts. A small group of ICAN researchers with expertise in qualitative methods (LS, EH, CB, SC, ZK) then queried code reports relevant to the constructs of Domain 1 of the Contraceptive Agency Framework (Table 1) and social influence on decision-making from Dedoose, drafted analytical memos, and identified key emerging themes. Themes and supporting excerpts were then mapped onto a matrix structured by specific Contraceptive Agency Framework constructs.

**Table 1:** Defining domain one of the Contraceptive Agency Framework: Agency in decision-making related to avoiding or delaying pregnancy^a^.

Construct	Definition
**Clarity of values**	To **be clear about one’s personal values** related to doing or not doing something to avoid or delay pregnancy
**Support and information one desires**	To **have information and support in accordance with one’s preferences** to make choices about doing or not doing something to avoid or delay pregnancy
**Consciousness of rights**	To be conscious of the right to contraceptive choice, including: • Entitlement to use, not use, switch, or stop using contraception • Right to have access to a wide range of contraceptive methods and to choose which method, if any, to use
**Critical reflection**	To **exercise critical reflection** and be aware that intersecting social constructions can constrain or enhance individuals' ability to exercise the right to choices related to doing or not doing something to avoid or delay pregnancy
**Perceived control**	To **believe one has control over decisions** related to doing or not doing something to avoid or delay pregnancy, including who they would like to be involved in those decisions
**Self-efficacy**	To **have confidence in one’s ability to form and act on preferences** related to doing something or not doing something to avoid or delay pregnancy

a The Contraceptive Agency Framework is grounded in interdisciplinary theories related to agency, critical consciousness, and health behavior. Definitions are from Holt et al. (2024).

After mapping preliminary findings to the individual constructs of the Contraceptive Agency Framework, the researchers met as a team to discuss and agree upon final themes. We have provided a more detailed description of the overall ICAN qualitative analysis process elsewhere (Suchman et al., 2023).

### 2.3 Ethical approvals

Ethical approval for the research was obtained from: the UCSF Institutional Review Board (270555, 270747, 270554, 270084); the Kenya Medical Research Institute’s Scientific Ethics Review Unit (KEMRI/SERU/CMR/P00136/4013) and Kenyan National Commission for Science, Technology and Innovation (464643); the Makerere University School of Public Health (SPH-812) and Uganda National Council of Science and Technology (HS1087ES); the Malawi University of Science and Technology Research Ethics Committee (P.03/2020/007); and the National Health Research Ethics Committee, Nigeria (NHREC 01/01/2007-25/09/2020).

## 3 Results

### 3.1 Participant characteristics

In our sample of 241 participants, 37 % were aged 15–19 and 63 % were aged 20–45; in total, 63 % had a history of contraceptive use (Table 2). Most participants had children with percentages ranging from 52 % of the sample in Nigeria to 75 % of the sample in Uganda. Overall, the sample consisted of 56 % married participants; these percentages were considerably higher in the Uganda sample (75 %) and lower in the Kenya sample (34 %). Employment status varied by study site, with the percentage of the sample employed ranging from 0 % in Malawi (where the sample was primarily subsistence farmers) to 73 % in Nigeria and Uganda. Finally, residence setting varied across study sites with the samples from Nigeria and Kenya being primarily from areas considered urban or peri-urban (76 % and 84 %, respectively), the sample from Malawi primarily from areas considered rural (87 %), and in Uganda the sample was evenly mixed between areas considered urban/peri-urban (47 %) and rural (50 %).

**Table 2:** Participants' demographic characteristics.

Demographic Variables		Kenya (n = 61)		Malawi (n = 60)		Nigeria (n = 60)		Uganda (n = 60)		Total (N = 241)	
**Age**		n	%	n	%	n	%	n	%	N	%
	15–19	25	41 %	18	30 %	16	27 %	29	48 %	88	37 %
	20–45	36	59 %	42	70 %	44	73 %	31	52 %	153	63 %
**Contraceptive use history**											
	Never used	25	41 %	22	37 %	20	33 %	23	38 %	90	37 %
	Prior or current use^a^	36	59 %	38	63 %	40	67 %	37	62 %	151	63 %
**Parity**^b^											
	Nulliparous	25	41 %	16	27 %	29	48 %	8	13 %	78	32 %
	Ever had children	35	57 %	43	72 %	31	52 %	45	75 %	154	64 %
**Marital status**^c^											
	Married	21	34 %	41	68 %	28	47 %	45	75 %	135	56 %
	Unmarried	40	66 %	19	32 %	32	53 %	11	18 %	102	42 %
**Employment status**^d^											
	Employed	22	36 %	0	0 %	44	73 %	44	73 %	110	46 %
	Unemployed	37	61 %	60	100 %^e^	16	27 %	14	23 %	127	53 %
**Residence**^f^											
	Rural	2	3 %	52	87 %	13	22 %	30	50 %	97	40 %
	Peri-urban	40	66 %	8	13 %	11	18 %	16	27 %	75	31 %
	Urban	11	18 %	0	0 %	35	58 %	12	20 %	58	24 %

a Contraceptive use was defined as using any effective method of pregnancy prevention, including so-called “traditional” methods such as withdrawal or fertility awareness methods.

b Missing data from Kenya (n = 1), Malawi (n = 1), Uganda (n = 7).

c Includes cohabitation; Missing data from Uganda (n = 4).

d Missing data from Kenya (n = 2), Uganda (n = 2).

e Sample from Malawi consisted primarily of subsistence farmers who did not describe themselves as formally employed.

f Missing data from Kenya (n = 8), Nigeria (n = 1), Uganda (n = 2).

### 3.2 Findings

Below, we characterize: 1) three key elements of the process of deciding whether or not to use contraception among our sample of women in four countries in sub-Saharan Africa; and 2) within each element of decision-making, how women’s ability to exercise different components of agency (bolded within the text) is constrained or bolstered. We highlight differences across countries and populations (e.g. married vs. unmarried) where appropriate. Our findings grouped by construct from the Contraceptive Agency Framework (Holt et al., 2024) are summarized in Table 3 and articulated in more detail below.

**Table 3:** Findings mapped to constructs from Domain 1 of the Contraceptive Agency Framework (Holt et al., 2024).

Findings				
		Women consider the dynamics of their closest relationships and how their individual choices about use or non-use would be received by significant people in their lives	Women weigh their values around contraception against the potential ramifications of a given decision to use contraception or not	Women draw on both internal and external resources to make an ultimate decision
**Contraceptive Agency Constructs**	**Clarity of values**		•Participants weighed the desire to prevent pregnancy with the tradeoffs/risks required to do so • Younger participants struggled with clearly defining their values related to contraception • Covert users exhibited high clarity of values	
	**Support and information one desires**	• For younger participants, mothers were highly influential		• Some participants sought support from partners in their decision making • Health care workers were regarded as reliable resources for contraceptive information • Younger women leaned on older female relatives for information and often did not trust the information that was shared by peers • Having information about contraception boosted self-efficacy and clarity of values, and supported agentive decisions
	**Consciousness of rights**	• Younger married participants had less consciousness of a right to contraceptive choice; older women with children frequently and clearly expressed their rights to the decision • Older, unmarried women expressed higher consciousness of their right to contraceptive choice	• Most participants felt they had a right to contraceptive use to protect their own health and their children’s health	
	**Critical reflection**	• Most participants accepted male authority over contraceptive decisions, rarely saw the contradiction in stating they have rights and then deferring to their husband		
	**Perceived control**	• Among married participants, perceived control over contraceptive decisions increased with age and parity • Older unmarried participants felt they had independent control over their own contraceptive decisions	• Young married participants felt pressure to have a first child, reducing their sense of control • Young unmarried participants often thought control over contraceptive decisions fell to their mothers	
	**Self-efficacy**			• Self-efficacy was bolstered by contraceptive knowledge • Participants with high levels of self-efficacy were especially equipped to handle situations where husbands disapproved of their desire to use contraception • Younger women were more likely to exhibit low self-efficacy and defer to others when making contraceptive decisions

When making decisions about contraceptive use:

1. Women consider the dynamics of their closest relationships and how their individual choices would be received by significant people in their lives.

Most women in our sample either directly consulted a close family member or friend about their potential contraceptive use, or thought carefully about what that person would say if they raised the topic of contraception. It was rare for women in our sample to consult no one, although the degree to which each woman described being influenced by those she consulted varied according to her own relative independence and how much control she believed she had over the final decision.

Married women typically discussed contraception with their husband and most married women desired joint decision-making with their spouse. They defined joint decision-making as having a discussion with their husband while recognizing that he had the final say. However across countries, many participants perceived their husband to be the “president” of the household (36-year-old married contraceptive user, Uganda) while it was their role to submit to his wishes. In Nigeria women also faced practical barriers to accessing contraception without spousal permission.

*Before [the provider] gave me [contraception], she asked me if my husband knows about what I am about to do? And I said yes. In some places, they make people bring their husbands before they are given the contraceptive*. (38-year-old married contraceptive user w/5 children, Nigeria)

Married women therefore had relatively little **perceived control** or, relatedly, **self-efficacy—**two components of what comprises agency in the contraceptive decision-making process. This was particularly true among younger married women, who were especially likely to cede control to husbands and sometimes in-laws who expect a first child to be conceived early in a marriage. These young women were more likely to lack a **consciousness of rights**—another component of agency in contraceptive decision-making.Many of these young women felt they had no right to make an independent decision specifically because they had married. The following married participant was indeed using contraception, but only because she had her husband’s approval:

*I am like a cow that has been bought, because they picked their wealth, and they used it to pay for my bride price. So, like a cow, they [my husband and his family] are the ones to determine when I should drink water, and when I should be given grass to eat. So, they have the complete right on whether I have children or not*. (17-year-old contraceptive user, Uganda).

For some older married women, particularly, those with several children, a right to further plan the family through making their own decisions on contraceptive use was more acceptable—as if they earned the right to choose contraception through the experience of childbirth and childrearing.

*I am always involved in making decisions about childbearing in our family. Am the one who carries the pregnancy, and I do experience all the pregnancy traumas by myself including the labor pains. So, I can say that I have the right to decide and be involved in childbearing decisions. And I also decide on when I can conceive the baby based on the age of my last-born child.* (26-year-old married non-contraceptive user w/3 children, Malawi)

Among all married women, regardless of age, it was rare for participants to exercise **critical reflection**—to recognize or question the larger social constructs that often gave rise to power imbalances in their relationships—or to acknowledge the contradiction in believing in their right to contraception while requiring “permission” to access it. In fact, some married women even took comfort in the dynamic of a relationship where contraceptive decisions were out of their control.

*I actually enjoy it because it gives me the assurance that someone has my back and he always looks out for me when he’s buoyant financially. I get whatever I want so am really ok with that arrangement ….Because he is the head in everything, irrespective of your contributions as the woman, he still should make the decisions.* (42-year-old married contraceptive user w/4 children, Nigeria)

On the other hand, it is worth noting that a number of women privileged the married pair as a critical unit that jointly makes decisions affecting the household, and we found this attitude to be most prevalent in Kenya and Malawi.

*[My husband] has a say because he is the one who gives me money for the injection, he tells me to go for the injection and he even reminds me when my appointment is due. He reminds me because he doesn’t want those little children to have a hard time.* (32-year-old married contraceptive user w/4 children, Kenya)

*I do not have [individual] power, but I decide together with my husband on how many children we want to have. So, we have power to decide as a family on the number of children we want to have and how we want to space them.* (19-year-old married contraceptive user w/2 children, Malawi)

Young, unmarried participants often reported that their mothers played a significant role in either supporting their contraceptive use or deterring it. Some mothers were very supportive and helped their daughters by taking them to the clinic to get contraception. Conversely, if these young women thought their mothers would disapprove, they were reticent to use, suggesting they felt a lack of **perceived control** over the decision of whether or not to use contraception.

*[My mother] used to say my child, me I have grown but you, you’re still young. So, I have to take you to learn about preventing pregnancies so that you can keep yourself for your future ahead to see how it will be.* (16-year-old married contraceptive user w/unknown number of children, Uganda)

*You know some parents are so bad. So, you get so scared that you cannot store [contraceptives] in the house*. (16-year-old single contraceptive user w/1 child, Kenya)

For older, unmarried women, many felt that being single afforded them independent rights to contraception if so desired, suggesting they had both more **consciousness of rights** and **perceived control** than the married women and younger, unmarried women in our sample.

*Because I’m not married yet, so I make my decisions by myself.* (22-year-old unmarried contraceptive user w/1 child, Nigeria)

2. Women weigh their values around contraception against the potential ramifications of a given decision.

When making a decision about contraception, women considered their desire to prevent pregnancy and why, and balanced this against potential tradeoffs they might have to make to get what they wanted. Tradeoffs ranged in severity from having an uncomfortable conversation with a spouse or parent to experiencing violence or being kicked out of their home. As consequences became more severe, a woman’s original reasons for wanting to use contraception diminished in importance in favor of placing more value on her own safety and survival. This active weighing of contraceptive use against other priorities and potential consequences illustrated **clarity of values**—another component of agency in contraceptive decision-making—among many women in our sample.

I: What might happen if you don’t follow [your husband’s] advice [regarding contraceptive use]?

*P: It can bring problems in our marriage and even lead to domestic violence […] my husband can become violent towards me […] it can even cause separation, my husband can chase me away.* (19-year-old married contraceptive user w/1 child, Uganda)

Regardless of whether or not they actually used contraception, nearly all of the women we interviewed across countries demonstrated **consciousness of rights** insofar as they had an underlying belief in women’s rights to their own bodies and to control their own health, which often prompted them to consider contraception in the first place. For example, married women often worried that another pregnancy might affect their health or the health of the children they already had.

*You need also to consider the age of the last-born child, and also need to give a woman the chance to fully recover. You can’t just be giving birth now and then each and every year. The body needs to recover and get back to its normal state. [ …] So, when making childbearing decision you need to consider the health of the other kids and the woman who carries the pregnancy.* (37-year-old married non-contraceptive user w/4 children, Malawi)

Older married women most often saw contraception as a tool to help maintain the physical and financial health of the family. In many cases, they were able to have a conversation with their husband in which they made this case and the couple came to an agreement, making it relatively straightforward for these women to choose contraception. Married women with disapproving husbands who chose to incur some degree of risk by using contraception covertly typically exhibited high **clarity of values** and **self-efficacy** to make a decision. Such women were able to articulate the importance of family planning for their particular situation–often citing maternal and child health needs or financial security–validating the rationale to go against their husband’s wishes.

*I have the right because he who wears the shoe knows where it pinches*. *If I say I should tell my husband, he may say no. So, as I have decided to do it without letting anybody know about it, is the best for me.* (24-year-old married contraceptive user w/4 children, Nigeria)

Younger married women, who had less **perceived control** in their relationships than their older counterparts, often felt pressure to have children because they were still young and felt they needed to meet social and familial expectations to “prove” their fertility. In cases where they did not yet have any children, these young women often chose not to use contraception because they felt this was not an appropriate choice until after having their first child. Sometimes, they also worried that using hormonal contraception would affect their ability to have children later when the time was right.

I: Can you tell me an occasion when you discussed birth control with your husband or mother-in-law?

R: It was when my mother-in-law was quarrelling that my sister-in-law had had only one child, so me who had been married with bride wealth should bear at least four or five children.

I: What would happen if you disobeyed their idea; for example, if they stopped you from using contraceptive and you went ahead and used it?

*R: They would be upset.* (20-year-old married non-contraceptive user w/1 child, Uganda)

While they felt they needed to give birth before using family planning, many of these younger participants also expressed a desire to establish financial security as their family grew. However, social pressure to have more children continued, and as a result, young married women often grappled with **clearly defining their values** related to preventing pregnancy. Faced with the dual pressures of ensuring their family’s well-being and meeting expectations to continue childbearing, many women chose to use contraception. This often meant hiding their use from disapproving extended family or, especially in Nigeria, defying their husbands. Though many young women approached this decision with **clear values**, their reflections revealed the difficulty of balancing these conflicting expectations.

*I feel happy [about preventing pregnancy], but if [my husband] knows, I won’t feel happy.* (19-year-old married contraceptive user w/1 child, Nigeria)

Young unmarried women faced conflicting social pressures as well. In our sample, these young women—typically nulliparous and living with their parents—worried about being kicked out of their homes if they didn’t have explicit permission to use contraception and their parents discovered their contraceptive use. On the other hand, these young women were also acutely aware of the stigma of adolescent pregnancy and worried about having to leave school, or face social censure for either being seen purchasing contraception or becoming pregnant.

*My father is a very tough man. He can kick me out of his house [if he finds me using contraception]*. (17-year-old unmarried non-contraceptive user w/0 children, Malawi)

*The young girls who use family planning most of the time they are judged when they line up for family planning.* (17-year-old married non-contraceptive user w/2 children, Kenya)

Young women were therefore stuck between potential punishment for using contraception and potential punishment for *not* using it and getting pregnant. This inherent contradiction made **clarity of values** particularly challenging for this population.

Older, unmarried women had fewer concerns about the potential repercussions of contraceptive use than their married and younger counterparts. Even if they were in romantic relationships, these women often made contraceptive decisions independently. However, like the younger unmarried women, these women were concerned with how a potential pregnancy might be seen by the community or how they could financially support a child as a single mother. They therefore exhibited **clarity of values** around balancing a potential pregnancy with social expectations and their own personal limitations.

*I felt that I was alone and having many children without a husband I felt I should do family planning because there was no one who would support me if I had many children and my income was also low*. (35-year-old widowed contraceptive user w/2 children, Kenya)

Given that there was less censure of older, parous women using contraception across countries, even if unmarried, there was less inherent contradiction in the decision-making process for these participants than for the younger, unmarried women in our sample.

3. Women draw on both internal and external resources to make an ultimate decision.

Some women find an easy path through the considerations above leading to an informed decision that aligns with their own values. For others, the process is complicated by conflicting pressures plus deficits in certain elements of agency, such as lack of **control** or **clarity of values**. In these instances, **self-efficacy** and support **and information to make a decision—**the final component of agency in contraceptive decision-making not yet discussed— can either boost (when present) or diminish (if lacking) a women’s agency in the decision.

Among married women who had trouble getting spousal support for their contraceptive use, some felt they could either use their own knowledge and **self-efficacy** or enroll others to make their case and get the outcome they wanted (**perceived control**, support **& information**). This was particularly notable in Malawi and Nigeria, where a number of participants reported involving family members or healthcare providers to educate their husbands about contraception and win their approval.

*The woman should discuss with the relatives of her husband. If the relatives are not helping her then she should go to the [community health worker] so that the [community health worker] should explain to the husband about contraceptives so that he should understand the advantages of using contraceptive methods.* (28-year-old married contraceptive user w/3 children, Malawi)

While all women regardless of age felt healthcare workers had the most trustworthy **information** about contraception, theoretically making them the preferred resource for many, younger women were often skeptical that healthcare workers could be trusted with confidentiality (lack of support **& information**) or might refuse to serve them at all due to their age.

*So, like if you go to the hospital and you are underage… like if you have not reached that age of starting to use things like that. Obviously, the doctor will deny you. Is it?* (17-year-old unmarried contraceptive user w/0 children, Kenya)

This skepticism around healthcare providers coupled with concern that peers could not provide reliable information made trusted older women all the more influential among young women when making decisions about contraceptive use.

*I get first from my friends, then I go to the elders, then I compare notes, but I usually follow the advice of the elders, because sometimes, some friends are not trusted, and so the advice they give you is also not to be taken seriously.* (18-year-old married non-contraceptive user w/1 child, Uganda)

Mothers and older female relatives (i.e. sisters, aunts) were often seen as a reliable source of support **and information** due to their experience with and knowledge of contraception. Leaning on older women helped young women find confidence to make contraception decisions; some fully deferred to the elders while for others the learning boosted their **self-efficacy** to make a decision.

*I will follow her advice because she is my mum, and she knows better than I do.* (19-year-old unmarried non-contraceptive user w/0 children, Nigeria)

*I also see that what [my mother] said was true, because I have seen some of these things happening to my own body. And I told her that, ‘Mother, what you have said is true. You have talked about an important issue.’ Even me I support going into family planning and I also feel my body.* (20-year-old unmarried non-contraceptive user w/2 children, Uganda)

## 4 Discussion

Our results show that contraceptive decision-making is a dynamic social process throughout which women’s agency may be supported or constrained at various points. When making decisions about whether or not to use contraception, women consider the opinions and desires of those closest to them, with most women consulting at least one other person regardless of their marital status. They weigh their personal values around contraception against the potential ramifications of a given decision and draw on both internal and external resources to make an ultimate decision. Women’s **perceived control** over whether or not they use contraception particularly stands out as a determining factor in the degree to which women have agency in the decision. In order to make an agentive decision, women first need to feel that they either have some level of control over the decision already, or that they can gain control through other means (e.g. enrolling support from the extended family or a close friend). While some women in our sample expressed having this control, many were constrained by patriarchal norms that: position men as the head of household and final decision-maker (Bikketi et al., 2016; Fadeyibi et al., 2022; Gardsbane et al., 2022; Mudege et al., 2017); stigmatize adolescent sexuality, and particularly adolescent girls' sexuality, and encourage both parents and community to police this sexuality (Atuyambe et al., 2005; Miller et al., 2021; Ninsiima et al., 2018; Wigle et al., 2022); stigmatize extramarital sexuality and childbearing (Jayaweera et al., 2018; Oluseye et al., 2024).

**Self-efficacy** and support **& information** were other agency-related constructs notable both for the complementary role they played to perceived control and to each other. Low information and support often aligned with low self-efficacy to make a decision, while high knowledge and support tended to result in high self-efficacy to make a decision. Further, when women had higher self-efficacy to make a decision, they were more likely to feel they could exert control in their relationships and over their ultimate decision to use contraception or not. Regardless of level of self-efficacy, many women expressed **clarity of values** when weighing the importance and relevance of the benefits of contraceptive use against potential consequences. They appeared to exercise agency when weighing different factors and deciding whether or not to use contraception. However, young unmarried women often faced conflicting values (social stigma around adolescent use of contraceptives versus stigma around adolescent pregnancy) and described struggling to decide how to navigate competing demands to come to an agentic decision about whether or not to use contraception.

**Consciousness of rights** might hypothetically play a role in boosting women’s decision-making agency in cases where they have little perceived control or self-efficacy. However, findings on this theme were equivocal in our sample. This was especially true among married women who often described having a right to control their own bodies, protect their health, and access information, but simultaneously conceded that men had the ultimate say in contraceptive decisions. The fact that the rights women believed they were entitled to in theory were at odds with the assertion that men were the heads of household and final decision-makers was typically accepted as the status quo. This disconnect was coupled with a virtually complete lack of expression of **critical reflection** among the entire sample; almost no one connected this power men have to their larger role in society and the patriarchal norms that discredit women’s decision-making. While patriarchal norms were almost always taken for granted, some women did find ways to achieve desired goals—whether it was overt/covert contraceptive use or a healthy, financially secure family—without disrupting such norms. These women demonstrate that it is possible to exert agency through prioritization of needs, even within a constrained context.

Additionally, we found that achieving **clarity of values** requires constant re-assessment, as women in different circumstances identified variations in power dynamics depending on factors such as age, marital status, and parity. During adolescence, young women must weigh the potential consequences of a pregnancy against the consequences of others discovering they are using contraception (and therefore sexually active). As they get older and marry, social expectations and husbands' desires around fertility often take precedence, potentially decreasing the importance of using contraception early on, while this importance increases once they have satisfied social expectations of the desired number of children and become concerned with preserving their own health and the health of their families. Each of these stages requires revisiting their own priorities and contextualizing these priorities within their current life conditions to reach a decision. Regardless of whether or not they ultimately choose to use contraception, their decision is the culmination of this balancing act to determine what fits best for them in the moment.

Despite many of the constraints on women’s agency we outline above, we see opportunities to support women in making agentive contraceptive decisions. In cases where women’s agency was constrained by structural factors at the level of the health system, such as perceived provider bias among young women, there are a number of potential solutions. These include interventions such as Beyond Bias, which has been shown to reduce provider bias related to provision of family planning services for young women and improve patient-centered care (Wagner et al., 2022). Other programs involve providers in developing solutions through human-centered design processes (Solo & Festin, 2019), work to align providers' personal values with their professional goals (O'Malley et al., 2021), and clarify provider values related to provision of care (Wollum et al., 2025).

At the interpersonal level, we note examples of women pushing against patriarchal norms in our own data and using “creative agency,” which Kawarazuka et al. (Kawarazuka et al., 2019) contend “may bring about subtle changes in personal power relations rather than radical changes in gendered structures.” For example, Kawarazuka et al. note that when a woman prioritizes cooking her husband’s lunch over caring for her crying baby, she may encourage her husband to feel kindly toward their child, and ensure her own continued access to precious resources. These subtle power shifts are exemplified in our data among the women that acknowledge the power their husbands have in decision-making yet still find a path to meet their own needs—sometimes by slowly coaxing their husband to see the benefits in contraception, or in other instances by having one child with the knowledge that establishing their fertility may grant them permission to plan the next birth through contraception.

Further, some programs aimed at dismantling aspects of patriarchy among male partners have demonstrated results. These include a program to educate men about the importance of participatory decision-making and provide a model for this decision-making in Uganda (Lecoutere & Wuyts, 2021) and numerous programs to increase male participation in caring for women’s reproductive health across sub-Saharan Africa (Manda-Taylor et al., 2017; Nkwonta & Messias, 2019). In most of these cases, an increase in support **& information** for men resulted in concomitant increases in contraceptive uptake and/or positive reproductive health outcomes for women. However, we note that such interventions often focus on men and do not always prioritize increasing women’s agency at the same time that men are empowered with involvement and information. This points to a need for future research to examine increases in women’s **perceived control** alongside male-targeted interventions to better understand how such interventions do or do not boost women’s agency. Given that few women in our sample exhibited critical reflection and the ability to recognize social constraints and injustices is a precursor to agency in decision-making (Watts et al., 2011), future programs should also consider ways to buildthis capacity.

Among younger, unmarried women some programs, such as Determined, Resilient, Empowered, AIDS-free, and Mentored lives (DREAMS), which aimed to increase condom use and reduce sexual risk among young women have shown promise (Floyd et al., 2022). DREAMS focused on increasing support **& information** among both young women and their communities, as well as **perceived control** among the young women themselves. However, it is important to note that structural factors such as poverty and social norms limited the study’s success. Given how ingrained patriarchal norms appeared to be in our own study findings, it is unclear if interventions that don’t specifically address these norms can ultimately be successful in both prompting women’s desire to exert more overt power in their relationships as well as their ability to do so.

## 5 Limitations

Although interview guides were designed to gather detailed information about women’s contraceptive decision-making, we note that there was limited depth in data collected on the decision *not* to use contraception among non-users in our sample. Our results are skewed towards a characterization of how women with experience using contraception navigated the decision of when to use contraception. Future research should explore more in-depth the decision-making process of those who have never used contraception. Further, we note that women in our sample did not articulate experiences of coercion, though this practice has been documented elsewhere in the literature (Grace & Anderson, 2018; Wood et al., 2022). It is possible that the sensitive nature of the topic precluded women from sharing instances where they felt they had no say in decisions related to contraception. To address these limitations and since it is challenging to collect nuanced data about sensitive and complex topics in cross-sectional interviews, we suggest future work in this area take an ethnographic approach to capture in more depth: 1) the ways in which decisions about contraceptive use and non-use are made and the context surrounding these decisions; and 2) the decision-making processes of those who do not want to use contraception (including users and non-users), and also those who are not using contraception but also not desiring pregnancy. Finally, a strength of our study is also a limitation. While conducting a multi-country qualitative study allowed us to achieve breadth in terms of data collected, summarizing our results across four countries required reducing nuance in the findings in order to meet the confines of a published manuscript. We have published more nuanced findings elsewhere (Birabwa et al., 2025; Gitome et al., 2025; Kamanga et al., 2025; Kwena et al., 2025; Sedlander et al., 2025) with additional manuscripts forthcoming. Interested readers may find the full list of publications from the ICAN Consortium on our website (https://ican.ucsf.edu/peer-reviewed-articles).

## 6 Conclusion

We conclude that women’s contraceptive decision-making is a highly complex process and this complexity may be the gateway to opportunity. Despite the many structural constraints on women’s agency, the shifting nature of power relations and agency over the lifecourse presents multiple points at which women can be approached and offered support for making their own decisions, in consultation with whomever they choose, related to contraception. However, it is clear from the existing evidence that individual agency is only one piece of the puzzle and that contextual factors, such as community support or investment in women’s health from a spouse, are also critical to lay the basis for truly agentive decision-making about whether or not to use contraception.

## CRediT authorship contribution statement

**Lauren Suchman:** Writing – review & editing, Writing – original draft, Visualization, Supervision, Project administration, Formal analysis, Conceptualization. **Emily Himes:** Writing – review & editing, Writing – original draft, Visualization, Formal analysis, Conceptualization. **Catherine Birabwa:** Writing – review & editing, Formal analysis. **Sneha Challa:** Writing – review & editing, Formal analysis. **Sarah Okumu:** Writing – review & editing, Formal analysis. **Zachary Kwena:** Writing – review & editing, Formal analysis. **Louisa Ndunyu:** Writing – review & editing, Project administration. **Serah Gitome:** Writing – review & editing, Project administration. **Pauline Wekesa:** Writing – review & editing, Project administration. **Mandayachepa Nyando:** Writing – review & editing, Project administration. **Martha Kamanga:** Writing – review & editing, Project administration. **Tamandani Jumbe:** Writing – review & editing, Project administration. **Innocencia Mtalimanja:** Writing – review & editing, Project administration. **Alfred Maluwa:** Writing – review & editing, Visualization, Conceptualization. **Lynn Atuyambe:** Writing – review & editing, Project administration. **Dinah Amongin:** Writing – review & editing, Project administration. **Betty Kaudha:** Writing – review & editing, Project administration. **Agnes Kayego:** Writing – review & editing, Project administration. **Ivan Idiodi:** Writing – review & editing, Project administration. **Chioma Okoli:** Writing – review & editing, Project administration. **Aminat Tijani:** Writing – review & editing, Project administration. **Ayobambo Jegede:** Writing – review & editing, Project administration. **Awawu Grace Nmadu:** Writing – review & editing, Project administration. **Shakede Dimowo:** Writing – review & editing, Project administration. **Janelli Vallin:** Writing – review & editing, Project administration. **Beth Phillips:** Writing – review & editing, Project administration. **Elena Sinha:** Writing – review & editing, Formal analysis. **Address Malata:** Writing – review & editing, Visualization, Conceptualization. **Elizabeth Bukusi:** Writing – review & editing, Visualization, Conceptualization. **Peter Waiswa:** Writing – review & editing, Visualization, Conceptualization. **Elizabeth Omoluabi:** Writing – review & editing, Visualization, Conceptualization. **Kelsey Holt:** Writing – review & editing, Visualization, Funding acquisition, Conceptualization.

## Funding statement

This research was funded by the 10.13039/100000865Bill & Melinda Gates Foundation (grant number OPP1216593).

## Declaration of competing interest

The authors declare the following financial interests/personal relationships which may be considered as potential competing interests: Kelsey Holt reports financial support was provided by 10.13039/100000865Bill and Melinda Gates Foundation. Elizabeth Bukusi reports a relationship with MSD 10.13039/100009947Merck Sharp & Dohme AG that includes: consulting or advisory, funding grants, and speaking and lecture fees. Elizabeth Bukusi reports a relationship with L'Oreal that includes: consulting or advisory and speaking and lecture fees. Elizabeth Bukusi reports a relationship with ANTIVA Biosciences that includes: funding grants. Ivan Idiodi, Chioma Okoli, Aminat Tijani, Ayobambo Jegede, Grace Nmadu, Shakede Dimowo, Elizabeth Omoluabi reports a relationship with Akena Plus Health that includes: employment. If there are other authors, they declare that they have no known competing financial interests or personal relationships that could have appeared to influence the work reported in this paper.
