# Corrigendum to “Opening the Black Box of agency in contraceptive decision-making: A cross-country qualitative study of a complex process” [SSM - Qualitative Research in Health (2025), (8C), 100615]^☆^

**DOI:** 10.1016/j.ssmqr.2025.100696

**Published:** 2026-06

**Authors:** Lauren Suchman, Emily Himes, Catherine Birabwa, Sneha Challa, Sarah Okumu, Zachary Kwena, Louisa Ndunyu, Serah Gitome, Pauline Wekesa, Mandayachepa Nyando, Martha Kamanga, Tamandani Jumbe, Innocencia Mtalimanja, Alfred Maluwa, Lynn Atuyambe, Dinah Amongin, Betty Kaudha, Agnes Kayego, Ivan Idiodi, Chioma Okoli, Aminat Tijani, Ayobambo Jegede, Awawu Grace Nmadu, Shakede Dimowo, Janelli Vallin, Beth Phillips, Elena Sinha, Address Malata, Elizabeth Bukusi, Peter Waiswa, Elizabeth Omoluabi, Kelsey Holt

**Affiliations:** a Institute for Health & Aging, University of California San Francisco, UCSF Institute for Health & Aging, Box 0646 490 Illinois St., Floor 12, San Francisco, CA, 94158, USA; b Department of Family and Community Medicine, University of California San Francisco, 995 Potrero Ave, San Francisco, CA, 94110, USA; c Makerere University School of Public Health, New Mulago Gate Rd, Kampala, Uganda; d Centre for Microbiology Research, Kenya Medical Research Institute, Mbagathi Rd, Nairobi, Kenya; e Department of Public Health, Maseno University, Busia Road Private Bag 407 05, Kisumu, Kenya; f Academy of Medical Sciences, Malawi University of Science and Technology, P.O. Box 5196, Limbe, Malawi; g Midwifery Department, Kamuzu University of Health Sciences, 52X8+782, Mahatma Gandhi Rd, Blantyre, Malawi; h AkenaPlus Health, 20F Road, Citec Estate, Along Ring Road, Mbora, Abuja, Nigeria; i Family Health Care Nursing Department, University of California San Francisco, 490 Illinois Street, San Francisco, CA, 94143, USA; j Departments of Global Health and Obstetrics and Gynecology, University of Washington, 1410 NE Campus Pkwy, Seattle, WA, 98195, USA; k Department of Obstetrics, Gynecology and Reproductive Sciences, University of California San Francisco, 1825 Fourth St., San Francisco, CA, 94158, USA; l Global Public Health, Karolinska Institute, Stockholm, 171 77, Sweden; m Busoga Health Forum, P.O. Box 805, Jinja, Uganda

## Abstract

The family planning field has historically focused on contraceptive uptake with less attention to women's agency in the contraceptive decision-making process. As a result, we know little about how women arrive at a decision to use contraception or not and why. We conducted a study to characterize: 1) key elements of the process of contraceptive decision-making among women in sub-Saharan Africa; 2) how women's ability to exercise agency in decision-making is constrained or bolstered throughout this process. Findings are drawn from 241 semi-structured, in-depth interviews conducted with women in Kenya, Uganda, Malawi, and Nigeria aged 15–45 who were both users (n = 151) and non-users (n = 90) of contraception. Data were collected from January–September 2021 and analyzed using a combined thematic and framework analysis approach guided by the Contraceptive Agency Framework. When making decisions about contraceptive use, women: 1) consider the dynamics of their closest relationships; 2) weigh their personal values around contraception against the potential ramifications of a given decision; 3) draw on both internal and external resources to make an ultimate decision. Throughout this process, women's perceived control over decision-making and self-efficacy to make and act on a decision stood out as critical to agency. Knowledge and support from partners and family members either bolstered or undermined women's confidence to decide on their own. The complexity of the contraceptive decision-making process and the ways in which priorities shift over women's lifespans offer multiple potential points for intervention to uphold women's agency.

The authors would like to apologize for any inconvenience caused.

